# P-742. Characterization of the Inoculum Effect in Battlefield Trauma Patients with Methicillin-Susceptible *Staphylococcus aureus* Infections

**DOI:** 10.1093/ofid/ofae631.938

**Published:** 2025-01-29

**Authors:** Despain Justen, Katrin Mende, Laveta Stewart, Matthew Igo, Ian Seibert-Parzyszek, M Leigh Carson, Wesley Campbell, Andrew Wyatt, David R Tribble, John L Kiley

**Affiliations:** USAF, San Antonio, Texas; Infectious Disease Clincial Research Program, JBSA Ft Sam Houston, Texas; Infectious Disease Clinical Research Program, Henry Jackson Foundation, Bethesda, Maryland; Infectious Disease Clinical Research Program, Bethesda, Maryland; Infectious Disease Clinical Research Program, Bethesda, Maryland; Infectious Disease Clinical Research Program, Department of Preventive Medicine and Biostatistics, Uniformed Services University of the Health Sciences, Bethesda, MD, USA, Bethesda, MD; Walter Reed National Military Medical Center, Bethesda, Maryland; Landstuhl Regional Medical Center, Landstuhl, Rheinland-Pfalz, Germany; Uniformed Services University of the Health Sciences, Bethesda, Maryland; BAMC, San Antonio, Texas

## Abstract

**Background:**

The clinical relevance of the cefazolin inoculum effect (CIE) in methicillin-susceptible *Staphylococcus aureus* (MSSA) isolates has been debated, with some studies showing poor outcomes in CIE(+) MSSA patients treated with β-lactams and others finding no effect. As less is known about CIE in trauma patients, we examined characteristics of US wounded military personnel with MSSA infections with and without CIE.

**Figure:**
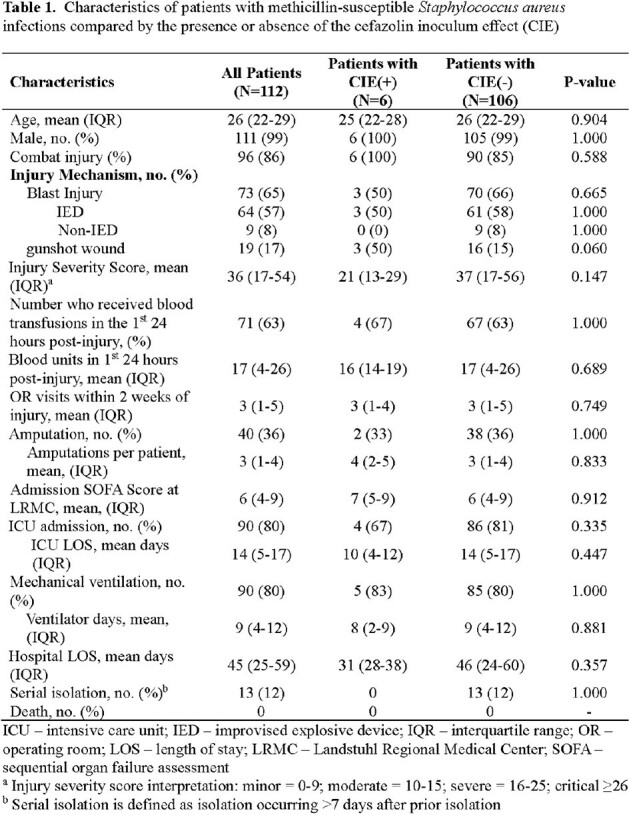

**Methods:**

Data and initial / serial (defined as isolation ≥ 7 days from prior isolate) MSSA isolates were collected from the Trauma Infectious Disease Outcomes Study (6/09-12/14). Isolates collected for surveillance were excluded. Antimicrobial susceptibility testing was performed via BD Phoenix Automated Microbiology System using CLSI criteria. Isolates were tested for CIE by broth microdilution utilizing standard (∼5x10^5^ cfu/mL) and high (HI) inocula (∼5x10^7^ cfu/mL). CIE(+) was defined as a MIC increase to ≥ 16 μg/mL with HI.

**Figure:**
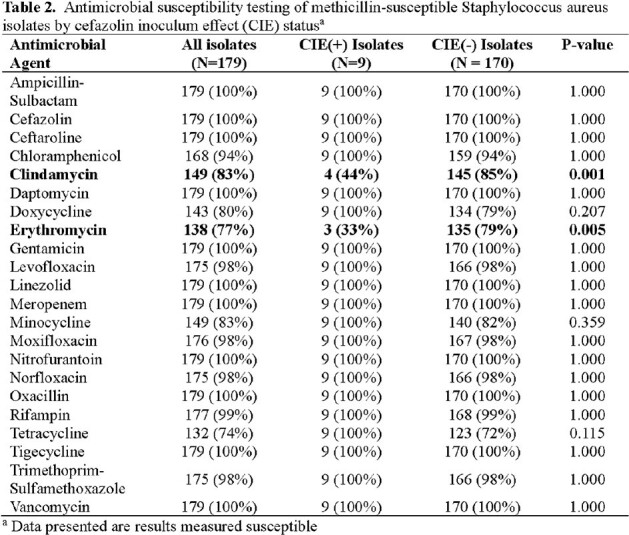

**Results:**

Among 112 MSSA patients, 6 had CEI(+) and 106 CIE(-) isolates. The patients were primarily males (99%) who sustained critical injuries (mean injury severity score 36) via blasts (65%), requiring critical care (80%) and prolonged hospitalization (mean 45 days, Table 1). All patients received 1^st^ generation cephalosporin or β-lactam with *Staphylococcus* coverage. There were no significant differences between the CIE(+) and CIE(-) patients. No CIE(+) patients had serial isolates, and no patients in either group died. A total of 179 MSSA isolates were assessed: 9 (5%) CIE(+) and 170 (95%) CIE(-). The majority of MSSA isolates were from respiratory (N=87, 49%), wound (N=72, 40%), and blood cultures (N=12, 7%). All isolates were uniformly susceptible to cefazolin, ampicillin-sulbactam, and ceftaroline, and had reduced susceptibility to doxycycline and tetracycline (Table 2). When compared to CIE(-) isolates, CIE(+) isolates had decreased susceptibility to clindamycin and erythromycin.

**Conclusion:**

CIE was uncommon with battlefield MSSA infections. Evaluation of clinical outcomes was limited (i.e., small numbers), but serial CIE(+) isolation was not observed. Although CIE(+) isolates were more resistant to clindamycin and erythromycin, isolates remained susceptible to other first-line antimicrobials.

**Disclosures:**

**All Authors**: No reported disclosures

